# “You Can See How Things Will End by the Way They Begin”: The Contribution of Early Mutual Obligations for the Development of the Psychological Contract

**DOI:** 10.3389/fpsyg.2018.00543

**Published:** 2018-04-17

**Authors:** Maria Luisa Farnese, Stefano Livi, Barbara Barbieri, René Schalk

**Affiliations:** ^1^Department of Psychology, Sapienza University of Rome, Rome, Italy; ^2^Department of Social and Developmental Psychology, Sapienza University of Rome, Rome, Italy; ^3^Department of Social Sciences and Institutions, University of Cagliari, Cagliari, Italy; ^4^Department of HR Studies, Tilburg University, Tilburg, Netherlands; ^5^Department of Economic and Management Sciences, North-West University, Potchefstroom, South Africa

**Keywords:** psychological contract, perceived mutual obligations, interaction, newcomers, longitudinal study

## Abstract

This study explores dynamic processes in the development of the psychological contract, focusing on the interaction of obligations related to the two parties (i.e., employees’ perceptions of both their own and the organization’s obligations fulfillment) on attitudinal outcomes (organizational commitment and turnover intention) during the initial stage of the employment relationship. In a twofold cross-sectional and two-wave study on newly hired correctional police officers, we examined: (a) whether perception of organizational obligations fulfillment moderates the relationship between employee obligations and their attitudes (Study 1, n.500); (b) the direct and moderated influence of perceived obligations at the entrance stage on those in the following months (Study 2, n.223). Results confirmed that, in the eyes of the newcomer, the obligations fulfillment of each of the two parties interact, having an additional effect beyond the main direct effects, in influencing both subsequent obligations perceptions and, through this, the outcome variables. Theoretical and practical implications of the findings are discussed.

## Introduction

The increasing environmental uncertainty, labor market mobility, and ongoing changes in organizational structures and work processes, impact the relationships between employees and organizations. Thus, the interplay between the perceived employees’ obligations toward the organization and *vice versa* (namely, the psychological contract) is an issue of renewed interest in contemporary employment relationships (Rousseau, 2001; Schalk and Roe, 2007; Coyle-Shapiro and Parzefall, 2008).

Grounded in social exchange theory (Blau, 1964) and based on its core elements—balance and reciprocity—scholars asserted that the fulfillment of obligations by the employer influences employee reactions (Levinson et al., 1962; Rousseau, 1990, 2001). Specifically, it is expected that employees tend to reciprocate the organization’s fulfillment of obligations by accordingly adjusting—by reducing or increasing—their own fulfillment of obligations toward the organization, in order to keep the balance in the employment relationship (De Vos et al., 2003; Schalk and Roe, 2007; De Vos and Freese, 2011). Hence, the interaction between the employer’s and the employee’s fulfillment of obligations captures the degree of reciprocity: in a balanced relationship, both the employer and the employee have similar levels of fulfillment (i.e., both high or both low), while in an unbalanced relationship one party is more fulfilled than the other (Shore and Barksdale, 1998; Payne et al., 2008). Overall, scholars have asserted that high balanced relationships are preferred and most desirable (Blau, 1964), and employees in balanced relationships are more likely to report positive organizational attitudes and behavior than employees in unbalanced relationships (Shore and Barksdale, 1998) and to maintain them over time (Robinson et al., 1994; De Vos et al., 2003; Ng and Feldman, 2008).

However, literature has neglected the reciprocity in fulfillment of obligations and has mainly focused on the consequences of psychological contract breach (e.g., absenteeism, lower trust) (Robinson, 1996; Turnley and Feldman, 1998, 1999; Lambert et al., 2003; Lo and Aryee, 2003; Coyle-Shapiro and Parzefall, 2008). Conversely, few studies have explored the underlying process that paves the way to these outcomes, through the concurrent contribution of the employee and the employer in building a balanced psychological contract (Coyle-Shapiro and Parzefall, 2008; Payne et al., 2008), or the development of this interplay during the time. This process is especially prominent in the early stages of development of the employment relationship among newcomers in the organization. For this reason, the present research focuses on the specific contribution of each of the two parties—as appraised by the newcomers—in fulfilling their obligations and explores the interactive influence of the fulfillment of both parties’ obligations on attitudinal outcomes (organizational commitment, turnover intention). Because the socialization period is a sensitive phase to shape the employees’ psychological contract (Nelson et al., 1991; Thomas and Anderson, 1998; Coyle-Shapiro and Parzefall, 2008), newly hired employees (specifically, correctional police officers in our two studies) were followed during their training—from early entrance (T1) to a later stage of socialization after their encounter with the operative environment (T2)—in order to examine the development of the psychological contract and the moderator role played by the fulfillment of the organization’s obligations.

This paper contributes to the literature in three ways. First (Study 1), by examining the concurrent interplay between perceived mutual obligations in the psychological contract and their relationships with employees’ attitudes (affective commitment, turnover intentions). Second (Study 2), by exploring whether and how this interactive process develops from the very beginning of the psychological contract to further stages of socialization. Third (Study 2), by examining how early perception of the organization’s fulfillment affects and shapes the individuals’ perceptions of their obligations and, in turn, their commitment. This research, highlighting the role of the perceived organization’s contribution, also provides new ways to develop practical suggestions for early intervention to enhance the development of an effective psychological contract.

### Psychological Contract and Work-Related Outcomes

The psychological contract is a mental model through which employees assess events happening at work. It is a main influencer of employees’ attitudes and behavior and can explain why employees adjust their attitudes and behavior in response to changes at work. The powerful influence of the psychological contract on outcomes such as commitment, organizational citizenship behavior, intention to stay, and attitude toward organizational change has been assessed in many studies (Turnley et al., 2003; De Vos and Meganck, 2008; De Jong et al., 2009; Alcover et al., 2012; Van den Heuvel et al., 2015). Most studies, however, focused on the fulfillment of organizational obligations by examining outcomes of psychological contract breach, such as reduced employees’ commitment, satisfaction, and performance (Knights and Kennedy, 2005; Tsui et al., 2013), lower organizational trust (Robinson, 1996; Pugh et al., 2003), decreased innovative and proactive work behavior (Ng et al., 2010; Bal et al., 2011), organizational citizenship behavior and voice (Turnley and Feldman, 1999; Coyle-Shapiro, 2002; Restubog et al., 2006, 2008), and higher organizational cynicism, turnover intention, and absenteeism (Johnson and O’Leary-Kelly, 2003; Addae et al., 2006; Deery et al., 2006; De Vos and Meganck, 2008). Also, meta-analyses focused on the negative effects of psychological contract breach of organizational obligations on work-related outcomes (Zhao et al., 2007; Bal et al., 2008; Topa et al., 2008).

Thus, although the literature widely acknowledged that the psychological contract is based on mutual obligations, most studies are on failure of organizational obligations. In this paper, we analyze the employee perception of the specific contribution of both parties in the psychological contract, as well as their influence on work-related outcomes. More specifically, we analyze the main effects of the fulfillment of both organizational and employee obligations, as well as their interactive effect. From an interactive perspective, employees’ perceptions of both their own and the organization’s obligations are relevant factors that affect the way the employment relationship develops, not only directly but also in a dynamic balance that metaphorically we might call a *pas de deux* (Anderson and Schalk, 1998; Dabos and Rousseau, 2004; Coyle-Shapiro and Parzefall, 2008). Since its early conceptualization, the psychological contract has been conceived as an interactive construct, whose essence lies in the interplay between both the employee’s and the employer’s perception about the employment relationship (Levinson et al., 1962; Schein, 1965). More recent conceptualizations emphasized the subjective perspective, proposing to take into account primarily the employee’s perception of both parties’ obligations (Rousseau, 1990; Freese and Schalk, 2008), highlighting the balance between the reciprocal obligations as a core process of the psychological contract. Although the psychological contract as a construct is clearly rooted in the social exchange theory framework (Blau, 1964), empirical research often did not fully capture this feature by focusing on the degree and quality of the delivery of the deal by the employer, while the interplay between the two parties’ obligations has been neglected.

Further, they mostly measured the employee’s holistic perception about the degree of mutuality, and only few researches examined the perceived specific contribution and the independent combination of both employee’s and employer’s obligations. Shore and Barksdale (1998) proposed four typologies of exchange relationships (degree of balance × level of obligation) and found that students perceiving mutual high obligations also showed higher levels of perceived organizational support, career future, affective commitment, and lower turnover intention. Dabos and Rousseau (2004), as well, found that mutuality—that is, the agreement regarding one party’s specific kind of contract (balanced, relational, transactional)—predicted specific outcomes (i.e., research productivity and career advancement). Measuring perceptions of the two parties’ contributions, De Jong et al. (2009) showed that high fulfillment of promises from both parties led to higher job satisfaction, fairness, and intention to stay. Payne et al. (2008) tested, in a longitudinal model, the direct and interactive effect of the two parties’ obligations on newcomers’ socialization activities. However, these authors did not find any direct effect and, contrary to their hypotheses, the significant interactive effects showed that those employees feeling that they were in unbalanced relationships (low in contributions and high in employer inducements) tried to rebalance them by spending more time with their mentor and in training. Overall, these results provided some empirical evidences to support Shore and Barksdale’s (1998) conceptualization, according to which mutual high obligations activate reciprocity and trigger a virtuous cycle, while mutual low obligations seem to determine a static and limited commitment from both parties. Less consistent are results related to unbalanced exchanges, which generally lead to medium levels of adjustment indicators, but further investigation is needed to better understand the specific contribution of each party in defining the unbalance and whether and how employees try to restore balance.

### The Psychological Contract’s Development

Dynamism is one of the main features of the psychological contract. It is assumed that the psychological contract can change over time in an adaptive process to preserve the balance of fulfillment of mutual obligations (Anderson and Schalk, 1998; Rousseau, 2001; Schalk and Roe, 2007; Coyle-Shapiro and Parzefall, 2008). Adopting a longitudinal perspective on the psychological contract, scholars have mainly focused either on its antecedents (i.e., factors causing changes in the psychological contract such as organizational support, leader-members exchange, socialization process, trust; Robinson, 1996; Dulac et al., 2008; Kiewitz et al., 2009) or on its effects on attitudes or behaviors (Restubog et al., 2006, 2008; Ng and Feldman, 2008; Payne et al., 2008; Conway et al., 2011; Delobbe et al., 2016).

Some researchers have analyzed the change of the psychological contract pattern during the development of the employment relationship, from the early stage to more enduring work relationships. For instance, Robinson et al. (1994) found that newcomers over time modified their perception of reciprocal obligations, showing increasing/decreasing patterns of fulfillment of some of their obligations toward the employer and decreasing patterns regarding fulfillment of some of their own obligations. Similarly, Thomas and Anderson (1998) confirmed that the newly hired recruits’ expectations on obligations that were more relational, long-term, and not job-specific (i.e., job security, social/leisure aspects, effects on family, accommodation) tended to increase. Ng et al. (2010) analyzed the effects of the psychological contract breach over a 6-month span and found that the extent of increase in psychological contract breach was significantly and positively related to the degree of decline in affective commitment and in innovation-related behaviors. However, to our knowledge, only a few scholars have more closely investigated the dynamic process of psychological contracts by examining the influence of early fulfillment of perceived obligations on perceptions in the aftermath.

Managing the psychological contract is especially important when introducing new hires in the organization (Rousseau, 1990; Thomas and Anderson, 1998; De Vos et al., 2003), since the 1st months after organizational entry are critical in shaping and stabilizing the psychological contract (Thomas and Anderson, 1998; Wanberg, 2012; Delobbe et al., 2016). In fact, the psychological contract can be conceived of as a sensemaking process, whose main function is reduction of insecurity by integrating all the issues that cannot be addressed in a formal, written contract thus both increasing the perceived predictability of organizational actions (Nelson et al., 1991; Shore and Tetrick, 1994; Coyle-Shapiro and Parzefall, 2008; Farnese et al., 2016) and helping newcomers to make early work-related expectations and beliefs become more realistic over time and bring them to reduce their feelings of unmet expectations or broken promises (Louis, 1980; De Vos et al., 2003; Sutton and Griffin, 2004; De Vos and Freese, 2011; Tomprou and Nikolaou, 2011).

The encounter stage is particularly important when taking into account the dynamics of psychological contracts (Anderson and Schalk, 1998) because in this period newcomers are more proactive in searching for additional information to define their psychological contract (De Vos et al., 2003; Dulac et al., 2008; De Vos and Freese, 2011). Once an individual’s schema is formed, it tends to crystallize (Coyle-Shapiro and Parzefall, 2008). The evaluation of the psychological contract during the initial employment stage is then a critical indicator of the way the relationship between the newcomer and the organization evolves over time, reflecting a process of positive or negative adjustment (Lance et al., 2000). Overall, scholars have suggested that newcomers’ experiences in this stage will influence not only organizational outcomes (e.g., motivation or commitment), but also the further development of their psychological contract (De Vos et al., 2003; Sutton and Griffin, 2004; Tomprou and Nikolaou, 2011; Wanberg, 2012). For instance, usually newcomers at first have idealized or poorly defined views of their employment relationship (Lance et al., 2000) and there are different patterns that can occur in the period after that, such as the honeymoon-hangover pattern (Boswell et al., 2009; Wang et al., 2017).

Although some longitudinal studies showed the impact of early psychological contracts on different outcomes, to the best of our knowledge only few studies provided empirical support to the pattern of evolution. Coyle-Shapiro and colleagues found support for the norm of reciprocity, showing that both perceived employer obligations and employer fulfillment of obligations at time 1 affected the employees’ fulfillment of obligations at time 2; conversely, employees’ fulfillment of obligations at time 1 affected the perceived employer obligations at time 2 (Coyle-Shapiro and Kessler, 2002). Authors confirmed these results in a further study (Coyle-Shapiro and Neuman, 2004). De Vos et al. (2003), in a longitudinal study on new hires in the encounter and acquisition socialization stage, found that the newcomers’ perceptions of their own contributions and of the inducements received by their employer influenced changes in perception of the promises they made to their employer and, conversely, the employer inducement that the newcomers perceived they had received influenced changes in their perception of the employer’s promises.

### Aim of the Studies

The first contribution of this research is to extend prior studies by confirming the relevance of psychological contract perceptions associated with both parties’ obligations, not only—as widely acknowledged—when the breach of promises leads to negative outcomes. Both parties obligations are needed to study the case of a ‘functional’ process. Specifically, we focused on the interplay between the employees’ perceptions of both their own and the organization’s fulfillment of obligations, assuming that an effective adjustment depends not only on what one party is perceived to do (employees’ contributions, organization inducements) but also by the moderating effect that the other party’s perceived obligations fulfillment exerts on these relations and on the related outcomes (affective commitment, turnover intention). Taking a social exchange (Blau, 1964) perspective, we expected that not only both parties’ fulfillment of obligations would influence attitudes (affective commitment, turnover intention), but also that there would be an interactive effect of the fulfillment of mutual obligations.

We further examined whether and how this interaction developed over time. To examine the interplay between perceived mutual obligations in its development from a sensitive phase of the employment relationship (that is the entry stage) to a later stage of socialization, we performed the second Study. It examined the associations over time by focusing on (a) whether the perception of the newcomer’s obligations in the early stage of socialization exerted an influence on the perception of later obligations, and (b) the interactive effect of the fulfillment of mutual obligations on subsequent outcomes. We supposed that newcomers’ initial perceptions of their obligations fulfillment would steer subsequent perceptions of the fulfillment of obligations and that this, in turn, would enhance the employee’s commitment. Moreover, we expected that this pattern would be shaped by the interpretation newcomers made about the organization’s fulfillment of obligations (De Vos et al., 2003). Based on the scarce empirical evidence available (Coyle-Shapiro and Kessler, 2002; De Vos et al., 2003), we could expect both linear effects (the employees’ fulfillment of obligations at time 1 enhances its development at time 2; and the same for perception of organization’s fulfillment) and interactive effects, related to a boosting effect of highly balanced contracts on their development, resulting in higher commitment.

## Study 1

**FIGURE 1:**
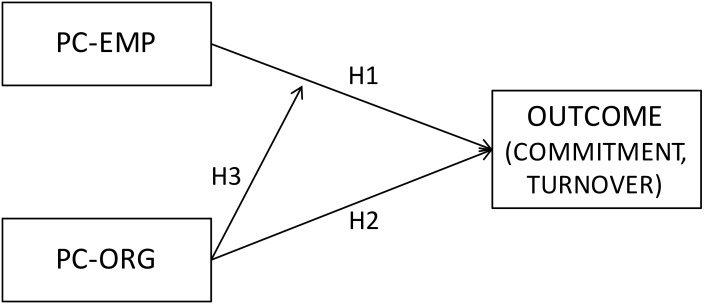
The theoretical moderated model of PC-EMP and PC-ORG on affective commitment and turnover intention.

The first aim of this paper was to analyze the specific contribution and the reciprocal moderator role of both fulfillments of obligations on attitudinal outcomes, examining whether the interaction between the organizational and employee obligations fulfillment could represent a factor that strengthens the effects of the fulfillment of obligations on subsequent attitudes, adding a specific effect beyond the main direct effects of the fulfillment by each party. Specifically we tested, on a sample of 500 newcomers, whether both perceptions of employees about their fulfillment of obligations toward the organization (H1) and perceptions about fulfillment of obligations by the organization (H2) exerted not only a specific and direct effect, controlling for the other factor, on employees’ attitudes (affective commitment and intention to quit)—but also an interactive effect, adding variance besides the explained variance of main effects (H3) (see **Figure 1**):

(H1) An employee’s perception of his/her fulfillment of obligations toward the organization will have a positive association with their affective commitment (H1a) and a negative association with their turnover intentions (H1b).

(H2) An employee’s perception of the fulfillment of the organization of the obligations toward them will have a positive association with their affective commitment (H2a) and a negative association with their turnover intentions (H2b).

(H3) The interaction of an employee’s perception of the organization’s fulfillment of obligations and the employee’s fulfillment of obligations will influence affective commitment (H3a) and turnover intentions (H3b) in such a way that the effects of high levels of fulfillment of both mutual obligations will have a stronger effect.

### Method

#### Participants and Procedure

Participants were correctional police officer cadets, hired with a permanent employment contract by the Ministry of Justice (Penitentiary Administration) and following a 12-month mandatory training, in order to work in prisons.

For Study 1, 519 cadets were asked to take part in the research when they were attending the 8th month of their course (T2), soon after a 2-month experience of stage in prisons (followed by a trainer). Questionnaires were administered during the training sessions, thus almost all cadets filled them in (about 97%). Due to the missing values from some participants, they were deleted and thus the final sample included 500 participants. As expected, most of them were males (56.2%) and young (mean age = 23.93, *SD* = 2.04, range = 19–29). About 95.3% of the sample had completed high school, while 1.0% had a university degree. For 9.3% of the respondents this was their first job and 25.2% had 1 year of previous work experience.

Each participant received, during the training sessions, a paper-and-pencil questionnaire and a presentation letter, containing a brief description of the research, its main objectives, and a guarantee for confidentiality of their responses. The Ethic Committee of the Department of one of the Authors approved the study.

#### Measures

*Psychological contract* was measured by assessing the newcomers’ perceptions about the Administration’s fulfillment of obligations and their own obligations fulfillment. In agreement with Freese and Schalk (2008), who argued that to measure the psychological contract a unilateral view is preferable (because the psychological contract is literally psychological, that is to say, it is by definition an individual perception), we used the scale by De Vos et al. (2003; Italian version, Barbieri et al., 2018), which takes into consideration the employee’s perceptions related to both parties’ obligations: the employee obligations (PC-EMP) and the organization obligations (PC-ORG). The PC-EMP is a 19-item scale focusing on five content dimensions of obligations (in- and extra-role behavior, flexibility, ethical behavior, loyalty, employability) and measuring the employees’ perceptions about the extent to which they fulfill these obligations. Example items are ‘Share information with your colleagues,’ ‘Work during the weekend if necessary,’ and ‘Follow the policies and norms of the organization.’ The PC-ORG is a 19-item scale focusing on five content dimensions of obligations of the organization (career development, job content, social atmosphere, financial rewards, work-life balance) and measuring the employees’ perceptions of the extent to which their organization fulfills these obligations. Example items are ‘Opportunities for promotion,’ ‘A good atmosphere at work,’ and ‘Respect for your personal situation.’ For both perspectives participants were asked to rate their agreement on a 5-point scale, from 1 (*totally disagree*) to 5 (*completely agree*). The PC-EMP and PC-ORG scales had a satisfactory reliability (respectively, α = 0.90 and α = 0.91).

*Affective commitment* was assessed using the six items composing the affective subscale of the Organizational Commitment Scale developed by Allen and Meyer (1990) translated in Italian by Pierro et al. (1992). This dimension refers to affective attachment to the organization, characterized by shared values, a desire to remain in the organization, and a willingness to exert efforts on its behalf (Mowday et al., 1982). Example items include ‘I do not feel “emotionally attached” to [this Administration]’ (reversed) and ‘[This Administration] has a great deal of personal meaning for me’. Participants were asked to rate their agreement/disagreement on 7-point scales, from 1 (*totally disagree*) to 7 (*completely agree*). The scale had a satisfactory reliability (α = 0.82).

*Turnover* captured intention to quit the prison Administration within the past month and was measured by a 4-item scale adapted from Sager et al. (1998) (e.g., ‘I frequently think about quitting my job’). Response choices ranged from 1 (*totally disagree*) to 5 (*completely agree*). The scale had a satisfactory reliability (α = 0.90).

#### Data Analysis

To test our hypotheses, in this study we used a moderated regression analysis using Hayes’ (2013) PROCESS macro (Model 1), which estimates simple slopes, a procedure preferable to conducting separate regression analyses at each level of the dichotomous variable.

As control variables, we used gender and age. In fact, some studies (see the meta-analysis by Bal et al., 2008) suggest that age has an effect on appraising the employment relationship and own opportunities to work in other organizations. Thus, we included this variable, although our subjects are very homogeneous from this point of view (they are all young and this is their first permanent employment contract).

### Results

**Table 1** shows the associations between the perception of organizational obligations fulfillment (PC-ORG), perception of employee obligations fulfillment (PC-EMP), affective commitment, turnover intent, age, and gender. As expected, the two fulfillments were positively correlated with each other and with affective commitment and negatively with turnover intention. Furthermore, affective commitment was negatively associated with turnover intention. Finally, age and gender (0 = male, 1 = female) did not show any significant correlation with the two fulfillments, whereas they were positively correlated with affective commitment, and negatively with turnover intent (only gender), although with a small effect size.

**Table 1:** Correlations, means and standard deviations for measured variables (*N* = 500).

	1	2	3	4	5	6
(1) Gender	–					
(2) Age	0.02	–				
(3) Affective commitment	0.12^∗∗^	0.11^∗^	–			
(4) Turnover intentions	-0.12^∗∗^	-0.05	-0.69^∗∗^	–		
(5) PC-ORG	0.01	0.07	0.48^∗∗^	-0.43^∗∗^	–	
(6) PC-EMP	0.02	0.05	0.50^∗∗^	-0.43^∗∗^	0.65^∗∗^	–
*Mean*		23.93	5.68	1.61	3.49	3.80
*Standard deviation*		2.04	1.04	0.83	0.65	0.61

*^∗∗^p < 0.01, ^∗^p < 0.05.*

We ran two regressions with affective commitment and turnover intention as the dependent variables. For each regression the predictors were PC-ORG, PC-EMP, and their interaction. Moreover, age and gender were added as covariates. Unstandardized coefficients are reported in **Table 2** (Aiken and West, 1991).

**Table 2:** Affective commitment and turnover intent regressed on the predictors (*N* = 500).

	Dependent variables			
	Affective commitment		Turnover intentions	
Predictors	*b*	*SE*	*b*	*SE*
Constant	4.36^∗∗^	0.47	2.10^∗∗^	0.39
PC-EMP	0.51^∗∗^	0.08	-0.34^∗∗^	0.07
PC-ORG	0.45^∗∗^	0.08	-0.33^∗∗^	0.07
PC-EMP ^∗^ PC-ORG	-0.18^∗^	0.08	0.15^∗^	0.07
Age	0.04^∗^	0.02	-0.01	0.02
Gender	0.22^∗∗^	0.08	0.19^∗∗^	0.07
*F*_(df)_	44.94_(5,494)_^∗∗^		32.27 _(5,494)_^∗∗^	
*R*^2^	0.31		0.25	

*^∗∗^p < 0.01, ^∗^p < 0.05.*

**FIGURE 2:**
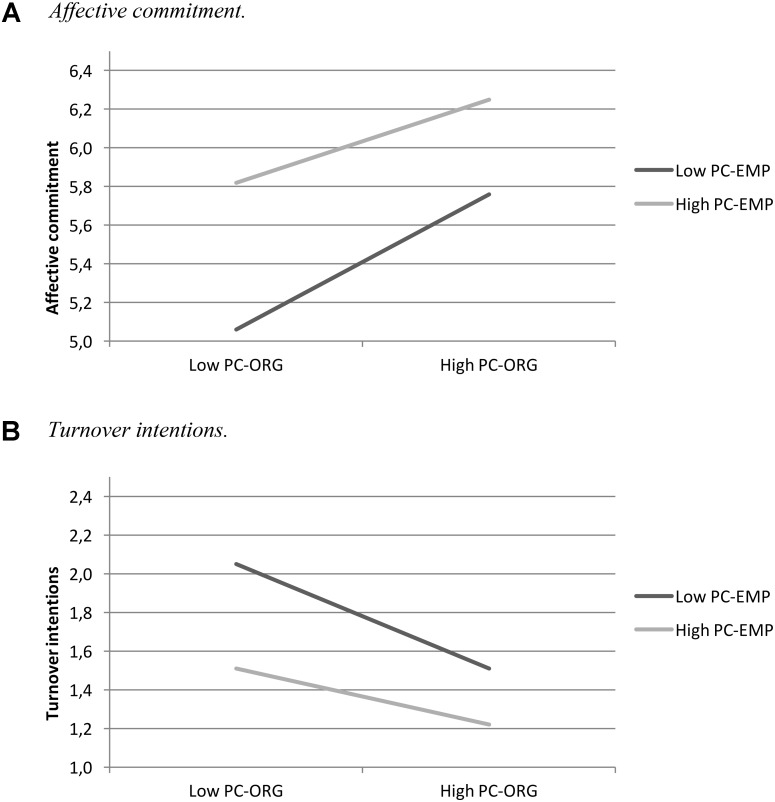
Simple slope analysis of the interaction between PC-EMP × PC-ORG on affective commitment **(A)** and on turnover intentions **(B)**.

When affective commitment was considered as the dependent variable, we found a strong regression weight both for PC-EMP (*b* = 0.51, *p* < 0.001) and for PC-ORG (*b* = 0.45, *p* < 0.001). This means that newcomers who feel motivated to fulfill their obligations are also more committed to the organization; similarly, those who feel that their organization fulfills its obligations toward them are more committed as well. More interestingly to our hypothesis, a significant PC-EMP × PC-ORG coefficient was found (*b* = -0.18, *p* = 0.029). Simple slope analyses (**Figure 2A**) revealed that, when the relationship between PC-EMP and affective commitment is positive—as demonstrated by the strong main effect—this effect is much stronger when the PC-ORG is high too (*b* = 0.56, *p* < 0.001). That is, the perception of the organizations’ obligations fulfillment generates an additive effect on commitment (Cohen et al., 2003).

With respect to turnover intent (**Table 2**), we detected a similar pattern with a significant main effect for the perceived employee (*b* = -0.34, *p* < 0.001) and the organizational (*b* = -0.33, *p* < 0.001) fulfillment of obligations. As for commitment, the PC-EMP × PC-ORG interaction was significant (*b* = 0.15, *p* = 0.026). Simple slope analyses (**Figure 2B**) revealed a stronger effect on turnover intention when employee fulfillment is low (*b* = -0.43, *p* < 0.001), rather than when it is high (*b* = 0.23, *p* = 0.003). Also in this case, an additive and protective effect on turnover occurs (Cohen et al., 2003), so when newcomers perceive mutual high obligations they show the lowest level of turnover intent.

Overall, Study 1 results show that when newcomers perceive that the organization reciprocates their obligations, this synergistically contributes to enhancing the relationship between the newcomers’ high fulfillment perceptions and their attitudes (commitment to the Administration or intention to stay). Thus, newcomers’ attitudes are not only the unilateral result of their perception about the degree each party fulfills its own obligations but also the reciprocal adaptation to their interpretation about the other party’s obligations fulfillment.

## Study 2

**FIGURE 3:**
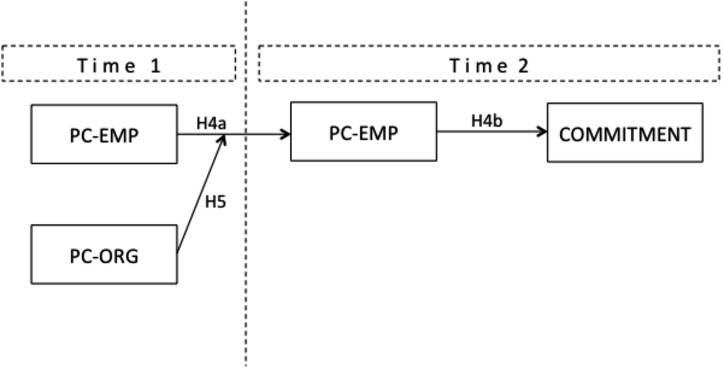
The theoretical longitudinal moderated mediation model of PC-EMP on affective commitment.

The first study allowed for verification of the spillover effects of fulfillment of mutual obligations and their interaction on commitment and turnover intention. In fact, the main result that emerged was that the fulfillment of individual obligations and those by the organization did interact beside the well-known main effects (Zhao et al., 2007; Bal et al., 2008; Topa et al., 2008). In particular we found that when the fulfillment of individual obligations is low, the fulfillment of obligations by the organization can ‘compensate’ by boosting commitment and reducing turnover intent. Taking into account the dynamic psychological contract conceptualization and research suggestions, our further aim was to test this interactive model in a longitudinal design, in a sample of newcomers. Thus, in Study 2 we analyzed data from a subsample of the previous study that was collected not only at the 8th month of training (T2) but also previously, at the beginning of their entry in the Justice Administration (T1, 3rd week). Specifically, we aimed to investigate whether newcomers’ adjustment of their attitudes (organizational affective commitment) could depend not only on the effect of the two parties’ fulfillment of obligations (Study 1) but also on their early perceptions about the two parties’ perceived obligations and their interplay. In a two-wave design, we tested whether and how the adjustment related to the two parties was rooted in the first employees-organizational contact, that is if: (a) the main effects did occur and develop also from newcomers’ original and idealized perceptions created at the very beginning of organizational entry (T1) to a more aware psychological contract (T2), in turn affecting their commitment (H4); (b) this process was shaped by the newcomers’ perception about the organizational fulfillment of obligations (H5, see **Figure 3**). Accordingly, we hypothesized:

(H4) A newcomer’s perception of his/her obligations toward the organization (PC-EMP) at T1 will influence their PC-EMP at T2 (H4a) that in turn will influence their organizational commitment (H4b).

(H5) The interaction of a newcomer’s perception of his/her obligations fulfillment and organizational fulfillment at T1 will influence affective commitment at T2. In particular, we expect that when both fulfillments are high, the perceived reciprocity will positively affect the PC-EMP T1/T2 relationship and, in turn, their affective commitment.

### Method

#### Participants and Procedure

Subjects considered for Study 2 were a subsample of cadets from the previous sample. Among the 519 cadets surveyed at the 8th month of training (T2), some of them also participated in the survey at the beginning of their entry into the Justice Administration (after they had attended about 3 weeks of training, T1)^1^. The questionnaire was administered to 224 cadets, who then took part in both the T1 survey and the T2 survey. Their characteristics were similar to those of the subjects composing the whole sample: males (50.7%), and young (mean age = 23.13, *SD* = 2.05, range = 19–28), 92.6% with a high school degree and 3.0% with a university degree.

#### Measures

We used the same scales used for Study 1: *Psychological contract* (De Vos et al., 2003) and *Affective commitment* (Allen and Meyer, 1990).

#### Data Analysis

To test the aforementioned hypotheses of mediation and moderated mediation, a regression analysis using bootstrapping resampling methods (1,000 bootstrap simulations) was conducted using PROCESS Model 7 (Hayes, 2013). We used the newcomers’ psychological contract at T1 (PC-EMP T1) as the independent variable, the newcomers’ psychological contract at T2 (PC-EMP T2) as the mediator, and the organizational psychological contract at T1 (PC-ORG T1) as the moderator. **Figure 2** displays the theoretical model we hypothesized.

The moderated mediation procedure split the model into two sub-models: the first analyses the main effects of the perceived obligations (PC-EMP and PC-ORG) and their interaction at T1 on fulfillment at T2, while the second model uses the perceived fulfillment of obligations as predictors and affective commitment as the criterion. As control variables, we used gender, age, and affective commitment at T1. The index of moderated mediation will be presented as well as the conditional effect of the mediator at values of the moderator.

### Results

Correlations, means, and standard deviations are shown in **Table 3**.

**Table 3:** Correlations, means and standard deviations for measured variables in Study 2 (*N* = 224).

	1	2	3	4	5	6	7	8
(1) Gender	–							
(2) Age	0.03	–						
(3) Affective commitment (T1)	0.01	0.11	–					
(4) Affective commitment (T2)	0.11	0.22^∗∗^	0.42^∗∗^	-				
(5) PC-ORG (T1)	-0.03	-0.04	0.29^∗∗^	0.19^∗∗^	–			
(6) PC-ORG (T2)	0.09	0.05	0.22^∗∗^	0.46^∗∗^	0.38^∗∗^	-		
(7) PC-EMP (T1)	0.03	0.06	0.44^∗∗^	0.29^∗∗^	0.59^∗∗^	0.27^∗∗^	–	
(8) PC-EMP (T2)	0.02	0.06	0.25^∗∗^	0.46^∗∗^	0.29^∗∗^	0.55^∗∗^	0.37^∗∗^	–
*Mean*	–	23.13	5.69	5.65	3.89	3.40	4.16	3.75
*Standard deviation*		2.05	0.98	1.05	0.49	0.62	0.49	0.59

*^∗∗^p < 0.01, ^∗^p < 0.05.*

The first part of the model—that used PC-EMP at T2 as the dependent variable—was statistically significant [*R*^2^ = 0.18; *F*_(6,217)_ = 7.87, *p* < 0.001] (see **Table 4**). Results show that PC-EMP at T2 was predicted significantly by PC-EMP at T1 (*effect* = 0.33, *SE* = 0.10, *p* = 0.001 [95% CI 0.14, 0.52]) (H4a), while the main effect of PC-ORG at T1 was not significant (*effect* = 0.13, *SE* = 0.09, *p* = 0.170 [95% CI -0.55, 0.31]). Also, all the covariates were not significant: age (*effect* = 0.01, *SE* = 0.18, *p* = 0.674 [95% CI -0.28, 0.43]), gender (0 = male, 1 = female) (*effect* = 0.12, *SE* = 0.07, *p* = 0.871 [95% CI -0.13, 0.16]), and affective commitment at T1 (*effect* = 0.61, *SE* = 0.04, *p* = 0.114 [95% CI -0.02, 0.14]).

**Table 4:** Results of the moderated mediation model in Study 2.

	Dependent variables			
	PC-EMP (T2)		Affective commitment (T2)	
Predictors	*b*	*SE*	*b*	*SE*
Constant	3.15^∗∗^	0.47	-0.88^∗∗^	0.81
PC-EMP (T1)	0.33^∗∗^	0.10	0.01	0.05
PC-EMP (T2)			0.66^∗∗^	0.11
PC-ORG (T1)	0.13	0.09		
PC-EMP (T1) ^∗^ PC-ORG (T1)	0.37^∗∗^	0.14		
Affective commitment (T1)	0.06	0.07	0.33^∗∗^	0.07
Age	0.01	0.02	0.08	0.03
Gender	0.01	0.07	0.19	0.11
*F*_(df)_	7.87_(6,217)_^∗∗^		22.86 _(5,218)_^∗∗^	
*R*^2^	0.18		0.34	

*^∗∗^p < 0.01, ^∗^p < 0.05*.

**FIGURE 4:**
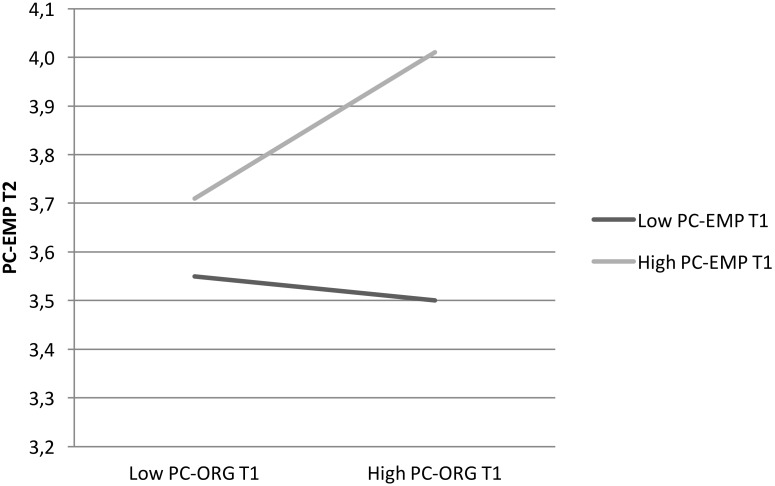
Simple slope analysis of the interaction between PC-EMP × PC-ORG at T1 on PC-EMP at T2 (*N* = 223).

Most importantly, the interactive effect of PC-ORG at T1 on the process from PC-EMP at T1 to T2 was significant (*effect* = 0.37, *SE* = 0.14, *p* = 0.009 [95% CI 0.09, 0.64]) (H5). In particular, as displayed in **Figure 4**, simple slope analysis reveals that when PC-ORG is high, the effect of PC-EMP at T1 on PC-EMP at T2 is significant (*effect* = 0.51, *SE* = 0.12, *p* = 0.001 [95% CI 0.26, 0.76]), whereas it becomes non-significant when PC-ORG is low. In other words, PC-EMP at T2 is especially strong when both PC-EMP and also PC-ORG at T1 are high.

Next, looking at affective commitment at T2 as the criterion, the model explained a significant proportion of the variance (*R*^2^ = 0.34; *F*_(6,218)_ = 22.86, *p* < 0.001). PC-EMP at T2 was significant (*effect* = 0.66, *SE* = 0.11, *p* < 0.001 [95% CI 0.45, 0.86]) (H4b), as well as the control variables: affective commitment at T1 (*effect* = 0.33, *SE* = 0.07, *p* < 0.001 [95% CI 0.20, 0.46]) and age (*effect* = 0.82, *SE* = 0.03, *p* = 0.004 [95% CI 0.03, 0.14]). Moreover, as in the previous model, gender was not significant (*effect* = 0.19, *SE* = 0.12, *p* = 0.105 [95% CI -0.04, 0.41]).

More importantly for our hypotheses, the index of moderated mediation for PC-EMP at T2 as mediator was significant (*index* = 0.24, *SE(Boot)* = 0.10, [95% Boot CI 0.06, 0.45]). In particular, as expected, the conditional indirect effect of PC-EMP at T1, through PC-EMP at T2 on affective commitment at T2, was significant only when PC-ORG at T1 was high (*effect* = 0.34, *SE(Boot)* = 0.10, [95% Boot CI 0.15, 0.55]), while it was not significant for the low level of PC-ORG. Hence, PC-EMP at T1 affects commitment at T2 only when PC-ORG at T1 is high.

Overall, results for this model show that newcomers low in PC-EMP at their entrance also remain low in the further stage of socialization (T2), whatever their perception about PC-ORG. Conversely, newcomers high in PC-EMP at their entrance subsequently increase their PC-EMP (T2) when they perceive that their organization is high in fulfilling its obligations. That is, when the organization reciprocates their obligations, this balance generates an additive effect that, in turn, leads to higher commitment. Thus, only mutual high obligations activate a virtuous cycle in psychological contract development.

### Discussion

This study aimed to explore the interplay between the two parties’ fulfillment of obligations (i.e., employee and organizational obligations and their influence on newcomers’ attitudinal outcomes) and the development over time from the entrance to the further acquisition socialization stage.

Results from the first study highlighted the interactive effect exerted by the perception of mutual obligations fulfillment: consistent with theoretical suggestions and sparse research findings (Shore and Barksdale, 1998; Dabos and Rousseau, 2004; De Jong et al., 2009) they showed that mutual high obligations led to the highest level of commitment, mutual low obligations to the lowest, and unbalanced psychological contracts to intermediate levels of adjustment (commitment, turnover intent). This result is in line with the norm of reciprocity assumption (Gouldner, 1960), according to which efforts from one party are reciprocated by the other to restore balance.

The second study proposed to examine newcomers’ psychological contract obligations development and their interaction during the socialization process. Also in this case, our findings suggested the development of a positive spiral of increasing promissory beliefs about both employee and employer obligations, which could indicate an unfolding relationship based upon reciprocity (Gouldner, 1960; Blau, 1964). In fact, in the case of mutual high obligations, the perceived organizational mutuality triggered a higher level of newcomers’ obligations in the following months and, in this manner, empowered their adjustment. Nonetheless, it is worth noting that under-obligations contracts (i.e., high obligations fulfillment by the employee and limited fulfillment by the organization) at the entrance stage did not seem to induce a further reduction of newcomers’ obligations. Similarly, in cases of over-obligations contracts, low motivated newcomers maintained low levels in the following months also when they perceived the organization’s fulfillment. Thus, results of the two-wave study showed rather independent development patterns for each of the two parties’ obligations unless, as for a ‘*pas de deux*,’ they did tune in on high expectations and fulfillment.

Overall, this study provided several contributions to the literature. First, it highlighted the importance of taking the independent and interactive contribution of each party into account, by not limiting its measurement to the holistic perception of the degree of balance (Freese and Schalk, 2008). Second, it suggested that the impact of each party’s contribution can change during the socialization process, and the subsequent pattern depends also on the balance achieved in the previous stages of socialization (Coyle-Shapiro and Kessler, 2002). This calls for further longitudinal studies on psychological contract development, taking into account both the different stages of socialization and the interplay between perceived mutual obligations, to provide a stronger evidence-based support. Third, this study delineated two patterns: a virtuous cycle between mutual high obligations, consistent with literature suggestions (Shore and Barksdale, 1998; Dabos and Rousseau, 2004; De Jong et al., 2009), where the feeling of being reciprocated by the organization’s obligations fulfillment enacts and empowers the employee’ fulfillment; and a ‘static’ pattern related to low employee’ obligations fulfillment. That is the case when newcomers low in psychological contract (regardless of high-low organizational fulfillment) tend to remain low. This means that, in the psychological contract process development, when newcomers are high in contract fulfillment they are sensitive to the organizational fulfillment and this can lead to higher mutuality conditions; conversely, when newcomers are low in contract fulfillment they seem to be insensitive to the organizational fulfillment, tending to not reciprocate. Future research should explore which factors, such as organizational socialization tactics (e.g., mentoring, proactive information seeking), shape the low pattern, making it possible to achieve a positive psychological contract.

#### Practical Implications

Understanding newcomers’ beliefs about the terms of their employment relationship is important from a managerial viewpoint since this will allow organizations, and especially human resource professionals responsible for recruitment and selection, to take into account and to actively manage the factors affecting employees’ perceptions of the terms of their psychological contract (De Vos, 2002). In line with social exchange theory (Blau, 1964), a mutual high fulfillment proved to be a key factor for activating a good psychological contract, which affects the way the employment relationship develops. In fact, our results clearly show that, in the eyes of the newcomers, individual and organizational psychological contracts do not work independently, but rather act together: hence it takes two to fulfill expectations. They further show that an early psychological contract contributes to influence its development. This means that organizational human resources policies are an important leverage for the employees’ management in both fulfilling their obligations consistent with the expectations of newcomers and favoring newcomers’ awareness about that. Moreover, these policies have to be handled early because, since the moment of the first entry, obligations fulfillment affects the future development of the contract. Considering the socialization span of time from early entrance to a later stage, Study 2 shows that only mutual high fulfillments enhance an effective adjustment process, whereas the possibility for the other part to restore an unbalanced psychological contract seems not to occur. Thus, future research should explore whether there are sensitive periods in the entrance stage when organizational programs could be more effective.

These findings are particularly useful in those careers that, although ensuring job security, do not offer professional growth prospects, are poorly paid, and often routinized. We are referring to professional careers inserted in organizational environments generally characterized by ‘high-demanding working contexts’ with high human and emotional density (e.g., nurses, teachers, correctional officers, etc.) and usually connected with phenomena such as turnover, work-related stress, and burnout (Schaufeli and Peeters, 2000; Farnese et al., 2017). In these work environments, as in Penitentiary Administration, most of the studies have focused on the significance of the psychological contract violation, paying attention primarily to the causes or the consequences of the psychological contract’s breach (Robinson, 1996; Turnley and Feldman, 1998, 1999; Coyle-Shapiro and Kessler, 2002; Lambert et al., 2003; Lo and Aryee, 2003), whereas the importance of the construction of a positive psychological contract has been extremely underestimated. According to Rousseau and Greller (1994), human resources processes and practices within organizations determine, to a large extent, the relationship between employer and employee. As a matter of fact, during the encounter phase newcomers experience the real demands at work, in exchange for rewards such as salary, promotions, and recognition (De Vos, 2002). This is the stage at which the psychological contract is formed and reliability is actively being tested (Nelson et al., 1991). A balanced psychological contract is necessary for a continuing harmonious relationship between the employee and the organization. This balance, especially during the early stage of entry, could serve as a protective function for the new employees, facilitating the development of positive attitudes (i.e., affective commitment, intention to stay).

#### Limitations and Further Research

A number of limitations of this study must be noted. First, we used self-report measures. Because we were primarily interested in newcomers’ perceptions and subjective evaluations of their employment relationship, the use of self-report data is justified (Wagner and Crampton, 1994). As discussed, this was felt to be the best method for assessing psychological contracts (Rousseau and Tijoriwala, 1998; Conway and Briner, 2002). However, this justification does not eliminate the potential problems of common method variance due to single-source bias and of socially desirable responding (Wagner and Crampton, 1994). Although the likelihood of common method bias was somewhat reduced by measuring independent and dependent variables at different points in time and by focusing only on the change portion of psychological contract measures (Cohen and Cohen, 1983), future research should supplement self-reports with data from supervisors, peers, or both. In addition, we used an obligations fulfillment measure, but we did not take into account the promises, which are the expectations that individuals have about the different psychological contract areas.

Furthermore, the sample involved in our study worked in a specific organizational context and all employees were placed with a permanent contract. For instance, it is plausible that the cadets perceive they have lack of job alternatives, and this could elicit different reciprocal reactions (e.g., withdrawal rather than intent to quit) to rebalance asymmetry in social exchanges (Turnley and Feldman, 1999). Moreover, the correctional officer job—because of the features depicted above—has low social desirability (Schaufeli and Peeters, 2000), and this could explain why low initial newcomers’ psychological contracts do not increase even when organizational psychological contract is high (employer over-obligation). Thus, the peculiarities of the context could limit the generalisability of our findings and have to be taken into account when interpreting our results. In future research, it would be appropriate to investigate organizations operating in different sectors or other types of formal employment contracts and include specific organizational factors that could affect the psychological contract development.

Finally, concerning limitations related to our research design, we are aware that the longitudinal research design is not without problems (Ployhart and Vandenberg, 2010). For instance, both stages we studied correspond to training. However, another important step for re-defining the psychological contract is when new agents start working in operational contexts (Institutes). Further, the two stages could be affected by the honeymoon-hangover pattern (Boswell et al., 2009; Wang et al., 2017), due to the 7-month time span and to the direct knowledge of the operational context that newcomers had during the stage (just before time 2). Thus, it would be appropriate in future research to do a longitudinal study with a larger number of observation times, in order to monitor the development of a psychological contract in a broader time frame.

## Ethics Statement

This study was carried out in accordance with the recommendations of ‘Comitato I.R.B. (Institutional Review Board), Department of Psychology, Sapienza University of Rome’ with written informed consent from all subjects in accordance with the Declaration of Helsinki. The protocol was approved by the ‘Comitato I.R.B. (Institutional Review Board), Department of Psychology, Sapienza University of Rome’.

## Author Contributions

MF designed and directed the research project. MF and SL conceived of the presented idea. MF and BB developed the theory. SL performed the analyses. RS contributed to results interpretation and to a critical revision of the paper. All authors discussed the results and contributed to the final version of the manuscript.

## Conflict of Interest Statement

The authors declare that the research was conducted in the absence of any commercial or financial relationships that could be construed as a potential conflict of interest.

## Footnotes

1 The Justice Administration manages training of new cadets in several schools located throughout the country. Subjects participating in the longitudinal study (Study 2, T1–T2, n.223) are those who attended the training course in that schools who took part in the research project from its beginning. Afterwards, cadets attending the training course in other schools (which took part later to the research project), added to them, thus composing the remaining part of the cross-sectional sample (Study 1, T2, n.500). All cadets were hired in the same public competition.
